# Supplementary material for: Cell Cycle Control by Optogenetically Regulated Cell Cycle Inhibitor Protein p21
Source: Biology (Basel). 2023 Aug 31;12(9):1194. doi: 10.3390/biology12091194 (PMC10525493; doi:10.3390/biology12091194)
Supplement: Supplementary file 1 [file biology-12-01194-s001.zip › biology-2559075-supplementary.pdf]

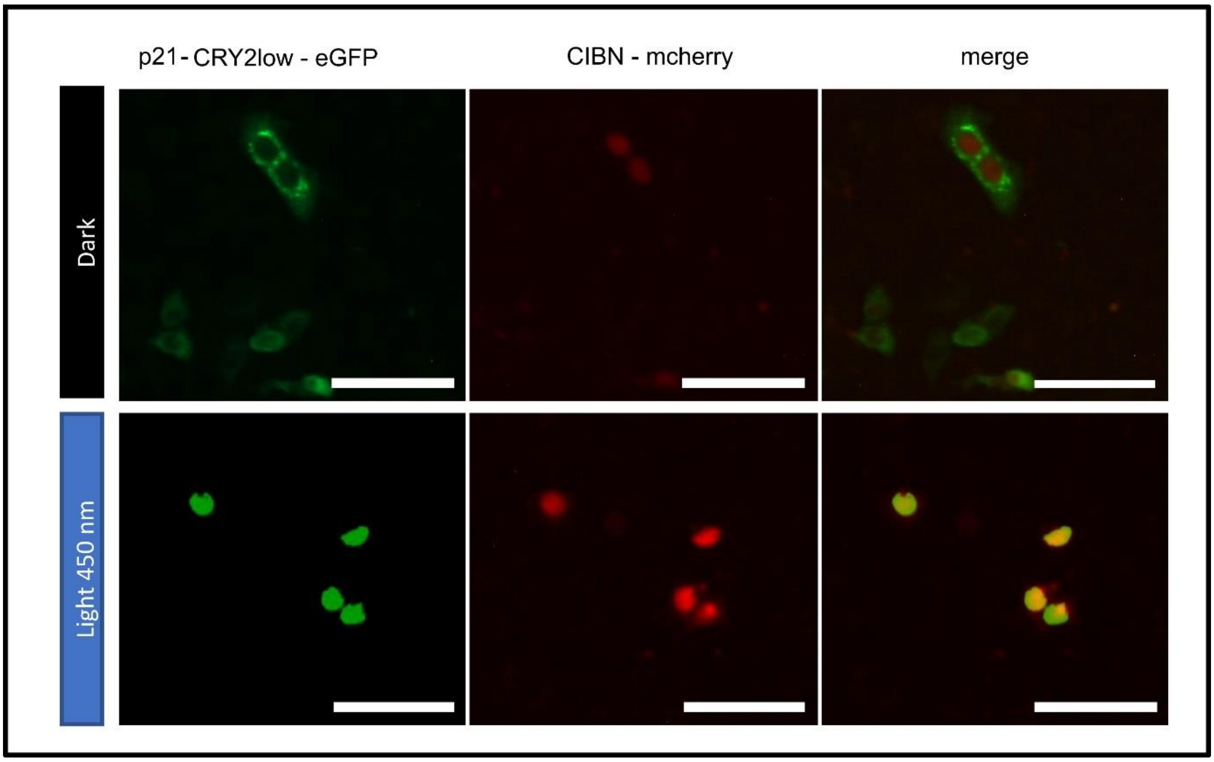


**Supplementary Figure S1.** Light-induced CRY2/CIBN nuclear translocation system. HEK293T cells were transiently cotransfected with HA-p21NLS$^{-}$-CRY2low-eGFP and CIBN-mCherry-HA (plasmid ratio 5:1). After incubation in the dark for 24 h, cells were either kept in the dark (upper panels) or illuminated (lower panels) with intervals of 2 s 450 nm blue light (1.33 W/m$^2$) and 3 min dark for 24 h. Cells were fixed with PFA and analyzed by fluorescence microscopy (EVOS FL microscope, 20x objective). Scale bars, 50 µm.

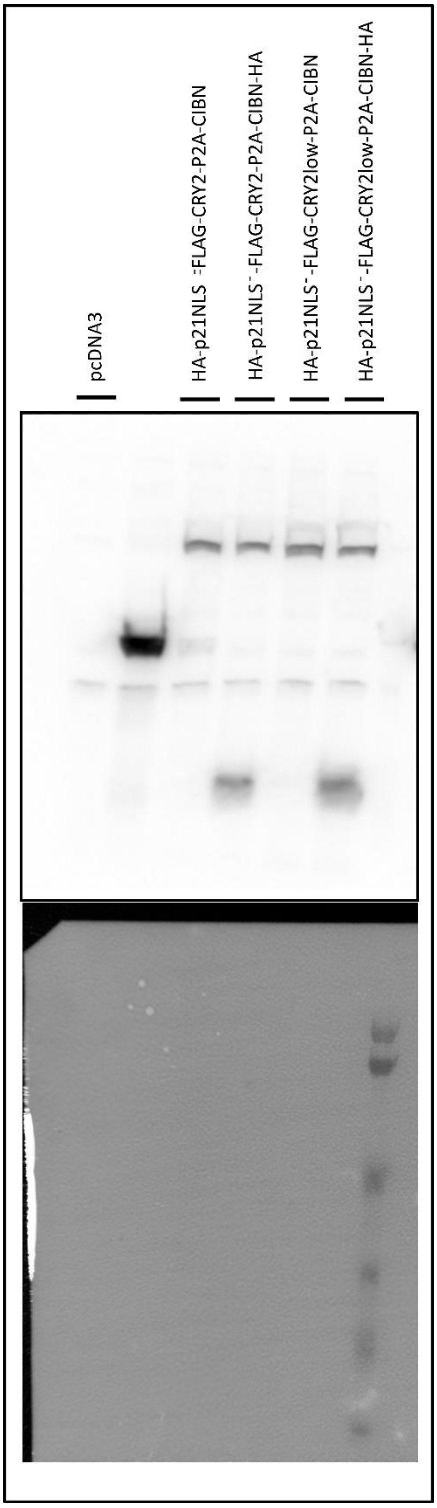


**Supplementary Figure S2.** Original Western blot image to Figure 1b and the corresponding PVDF membrane showing the protein molecular weight markers.

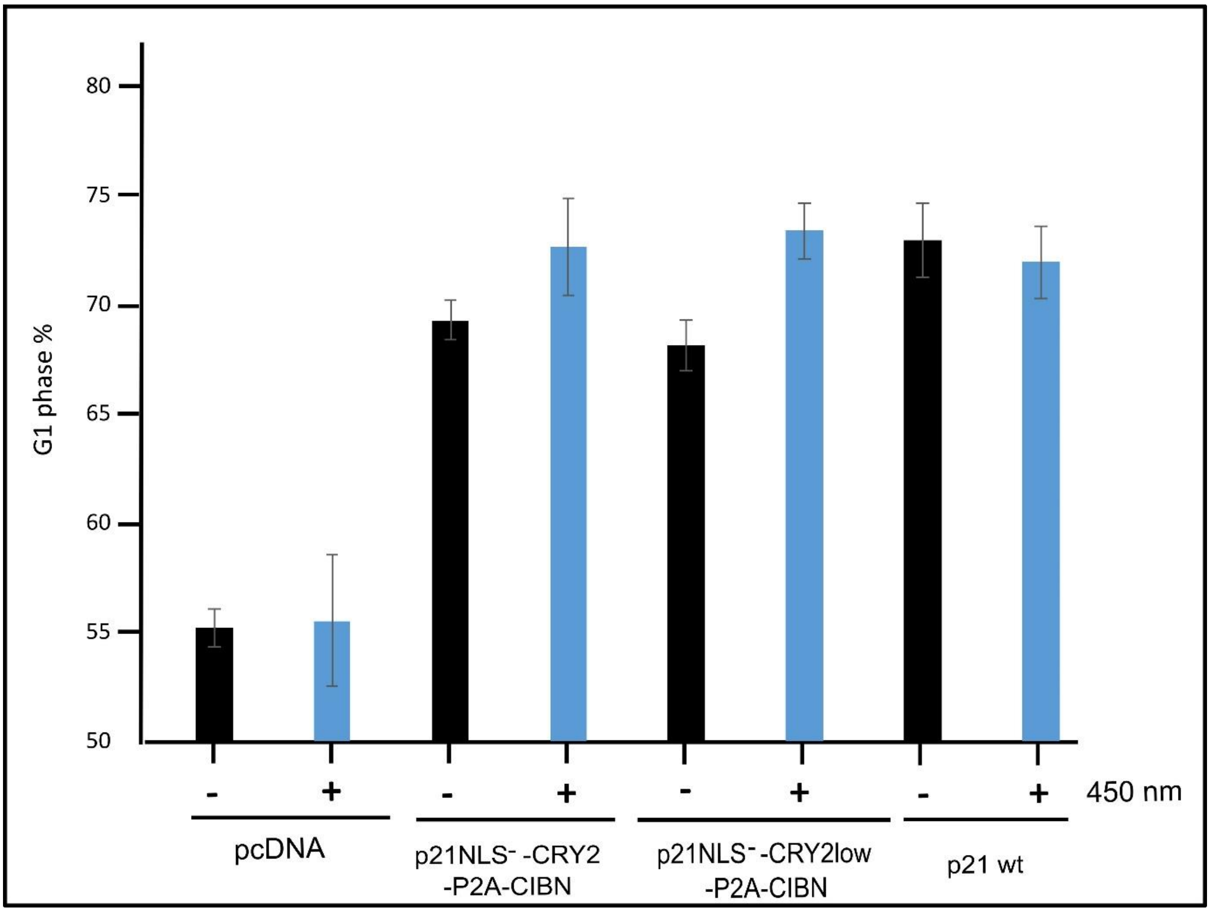


**Supplementary Figure S3.** G1 cell cycle arrest induced by light-regulated p21 and by wildtype p21. HEK293T cells were transfected with the constructs indicated. 24 h after transfection, the cells were illuminated with intervals of 2 s blue light (1.33 W/$m^2$) and 3 min dark for 24 h. Afterwards, the cells were fixed with ethanol and treated with propidium iodide to determine the distribution of the cell cycle phases with and without blue light illumination by FACS analysis. The cell cycle distribution was determined with the help of the Michel H. Fox algorithm. Percentile of cells arrested in the G1 phase are shown. The error bars indicate the standard deviation of three wells.

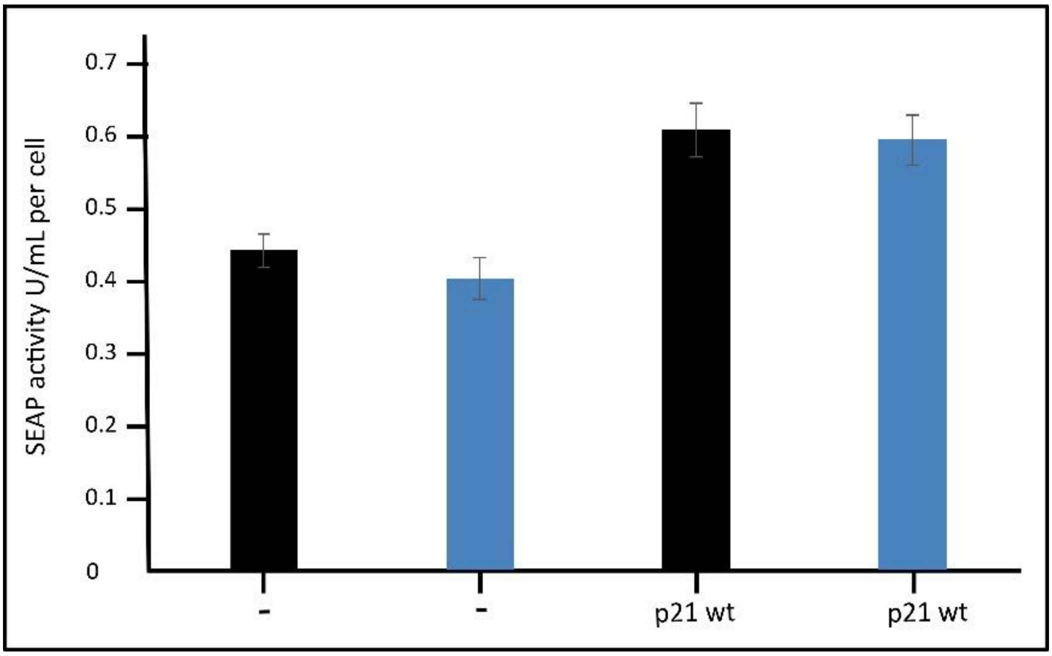


**Supplementary Figure S4.** Influence of wildtype p21 on cell-specific SEAP reporter activity. CHO cells were cotransfected with a wildtype p21 construct (pcDNA3-p21 wt) and the SEAP reporter plasmid or the SEAP reporter plasmid alone (-) as control. 10 ng DNA of each plasmid was used and the sample was adjusted to a total of 100 ng DNA by pcDNA3. 24 h after transfection, the cells were illuminated with intervals of 2 s blue light (1.33 $W/m^2$) and 3 min dark for 48 h. After the illumination the supernatant was analyzed for SEAP activity and the cell count was determined with an CASY counter to calculate the SEAP activity per cell. The error bars indicate the standard deviation of three 6 wells.

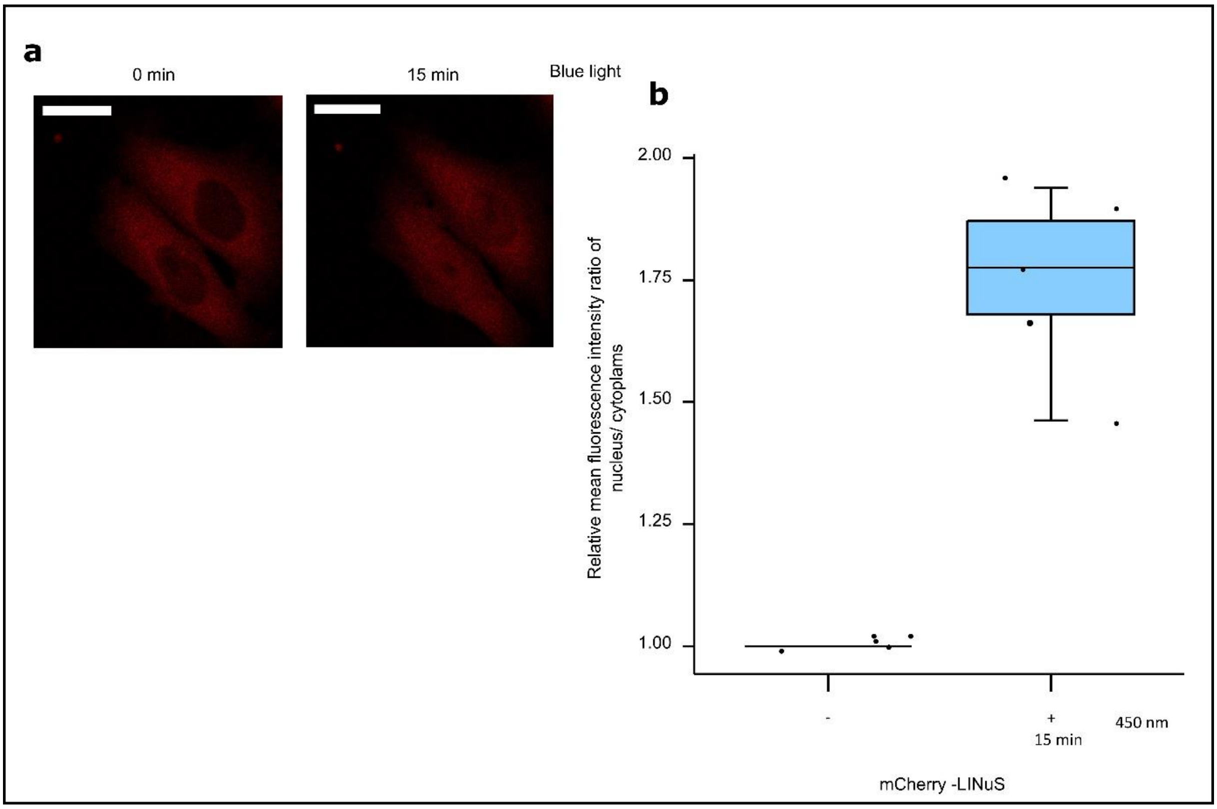


**Supplementary Figure S5.** Light-induced translocation of LINuS (mCherry-NES-AsLOV-NLS) from the cytoplasm into the nucleus. (a) Live cell imaging of CHO cells expressing LINuS for 24 h and subsequently kept in the dark or illuminated with blue light (1.33 W/m$^2$) for 15 min. Scale bars, 20 μm. (b) Quantification of the ratio of the relative mean fluorescence intensity of cytoplasmic and nuclear mCherry in CHO cells expressing LINuS after 15 min blue light illumination. Each circle represents one cell (n =5) and the ratios were normalized to the dark state of the cell.

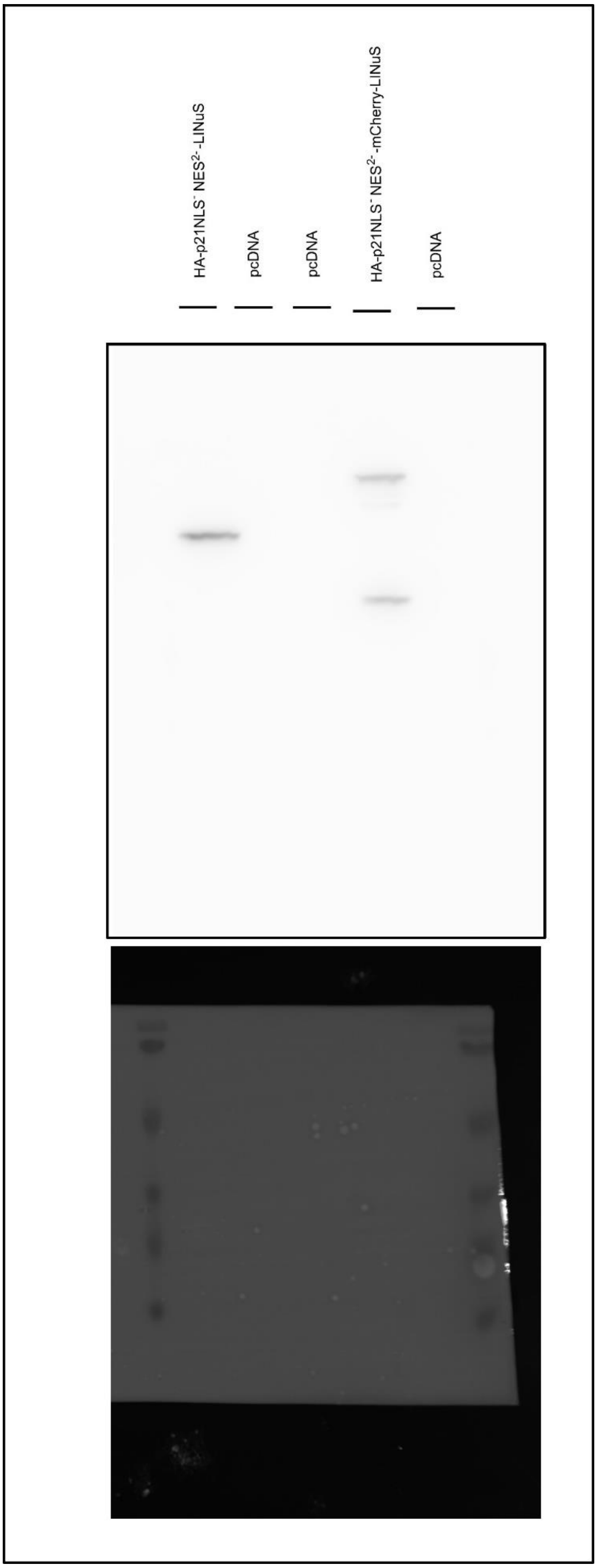


**Supplementary Figure S6.** Original Western blot image to Figure 4b and the corresponding PVDF membrane showing the protein molecular weight markers.

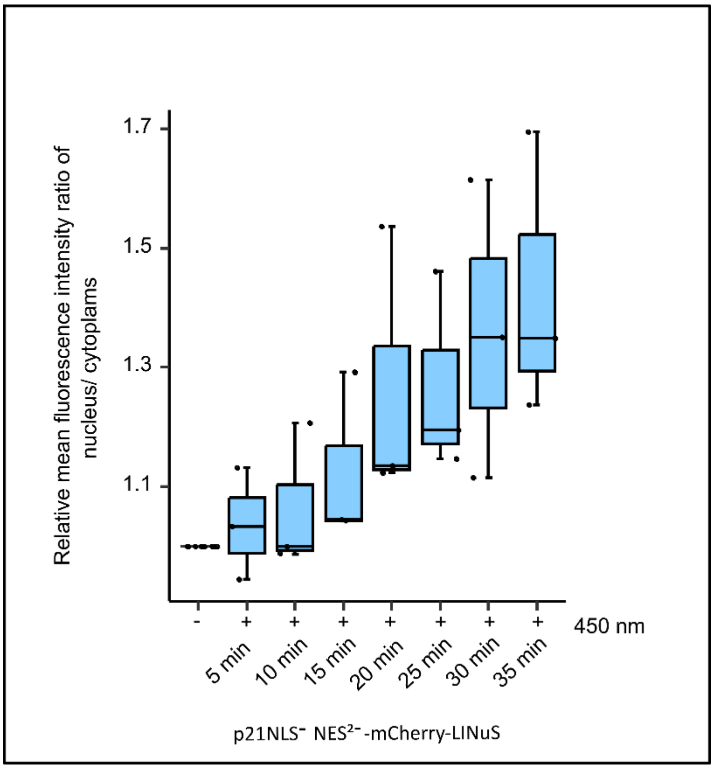


**Supplementary Figure S7.** Light-induced translocation of p21-LINuS from the cytoplasm into the nucleus. Quantification of the relative mean fluorescence intensity ratio of cytoplasmic and nuclear mCherry in CHO cells transfected with p21 $NLS^{-}NES^{2-}$ mCherry-LINuS after blue light illumination for up to 35 min. Each circle represents one cell (n = 3) and the ratios were normalized to the dark state of the cell.

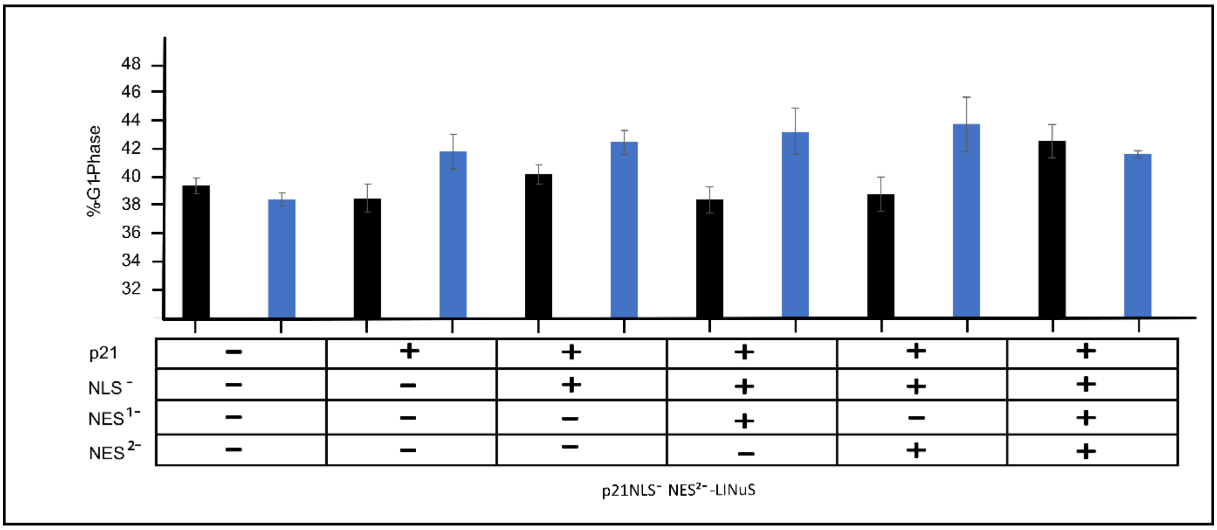


**Supplementary Figure S8.** Light-controlled G1 cell cycle arrest induced by p21-LINuS. CHO cells were transfected with p21NLS$^-$-NES2$^-$-mCherry-LINuS. 24 h after transfection, the cells were illuminated with intervals of 1 s blue light (1.33 W/m$^2$) and 30 s dark phase for 48 h. Afterwards, the cells were fixed with ethanol and treated with propidium iodide to determine the distribution of the cell cycle phases with and without blue light illumination by FACS analysis. The cell cycle distribution was determined with the help of the Michel H. Fox algorithm. Percentile of cells arrested in the G1 phase after blue light illumination are shown. The error bars indicate the standard deviation of three wells.

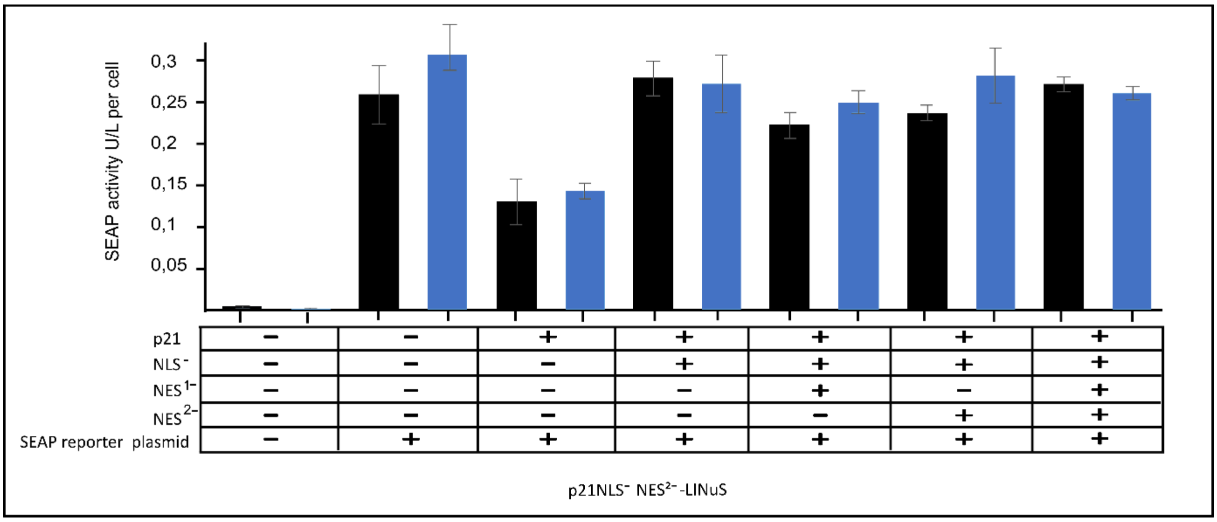


**Supplementary Figure S9.** Cell-specific SEAP activity regulated by p21-LINuS. Cells were cotransfected with the plasmid p21NLS$^-$-NES2$^-$-mCherry-LINuS and the SEAP reporter plasmid or the SEAP reporter plasmid alone as control. 24 h after transfection, the cells were illuminated with intervals of 1 s blue light (1.33 W/m$^2$) and 30 s dark phase for 48 h. The error bars indicate the standard deviation of six wells.
